# Performance Evaluation and Micro-Mechanisms of Composite Asphalt Modified by Desulfurized Rubber Powder and Distinct Waste Plastics

**DOI:** 10.3390/polym18080973

**Published:** 2026-04-16

**Authors:** Dongwei Cao, Mingming Zhang, Rui Zheng, Qidong Su, Wenbo Zhou

**Affiliations:** 1 School of Materials Science and Engineering, Chang’an University, Xi’an 710064, China; caodw_paper@163.com; 2 School of Highway, Chang’an University, Xi’an 710064, China; 3 CTVIC, Research Institute of Highway Ministry of Transport, Beijing 100088, China; 4 School of Civil Engineering, Hebei University of Engineering, Handan 056038, China; 5 School of Transportation, Southeast University, Nanjing 211189, China

**Keywords:** waste plastics, desulfurized rubber powder (DRP), composite-modified asphalt, rheological properties, microscopic mechanism, pavement performance

## Abstract

The synergistic utilization of waste plastics and tires in asphalt modification is a highly promising sustainable strategy. However, the differential impacts of distinct plastic molecular architectures on the performance and network evolution of rubber-modified asphalt remain fundamentally unclear. This study systematically investigated the physical, rheological, and microstructural properties of composite asphalts modified with desulfurized rubber powder (DRP) and four representative plastics: polyethylene (PE), styrene–isoprene–styrene (SIS), styrene–ethylene–butylene–styrene (SEBS), and styrene–butadiene–styrene (SBS). Furthermore, the pavement performance of the asphalt mixtures prepared via dry and wet methods was comparatively evaluated. Microstructural and spectroscopic analyses revealed that the composite modification was primarily governed by physical blending and swelling. The non-polar, semi-crystalline PE resulted in severe phase separation and extreme low-temperature brittleness. Conversely, the saturated hydrogenated mid-blocks of SEBS endowed the asphalt with the highest high-temperature rutting resistance but severely compromised its low-temperature stress relaxation. Remarkably, SBS interacted synergistically with DRP to form a highly homogeneous and densely interwoven three-dimensional network, thereby achieving an optimal viscoelastic balance, outstanding storage stability, and superior low-temperature ductility. Pavement performance tests further demonstrated that the wet method significantly outperformed the dry method for block copolymers by facilitating sufficient pre-swelling. Overall, the SBS-DRP composite-modified asphalt prepared via the wet method exhibited the most exceptional and balanced comprehensive pavement performance, providing a robust theoretical foundation for the sustainable and high-value recycling of multi-source solid wastes in paving engineering.

## 1. Introduction

The rapid expansion of global transportation infrastructure, coupled with increasing traffic volumes, heavier axle loads, and extreme weather conditions, has imposed increasingly stringent performance requirements on asphalt pavements [1]. To meet these rigorous demands and mitigate various pavement distresses, a diverse array of functional additives is routinely incorporated into the base asphalt. Generally, these additives can be categorized based on their primary purposes: polymer modifiers (such as elastomers, plastomers, and reactive resins) are utilized to enhance the viscoelastic properties, temperature susceptibility, and overall rutting resistance of the asphalt; adhesion additives (anti-stripping agents) are applied to improve the interfacial bonding between asphalt and aggregates to resist moisture damage; and aging inhibitors (antioxidants) are introduced to retard oxidative degradation and extend the service life of the pavement [2,3]. Among these, polymer modification has proven to be an exceptionally effective and widely adopted strategy for upgrading the comprehensive performance of asphalts. Concurrently, the exponential accumulation of solid wastes, particularly waste tires and waste plastics, poses an unprecedented threat to the global ecological environment and human health [4,5]. Disposing of these non-biodegradable polymeric wastes via conventional landfilling or incineration not only occupies vast land resources but also generates severe secondary environmental pollution and massive greenhouse gas emissions [6]. Therefore, developing eco-friendly and sustainable strategies to recycle these polymeric solid wastes into pavement engineering materials has emerged as a highly promising “waste-to-wealth” approach [7,8]. This strategy not only effectively mitigates environmental burdens but also substantially reduces the highway industry’s reliance on non-renewable fossil-based asphalt resources, aligning perfectly with the global goals of carbon neutrality and sustainable development [9,10].

Crumb rubber-modified asphalt (CRMA) has been extensively investigated and is widely recognized for its excellent high-temperature rutting resistance, prolonged fatigue life, and superior noise reduction capabilities [11,12]. However, the primary challenge for CRMA lies in the poor thermodynamic compatibility between the vulcanized, three-dimensional cross-linked rubber particles and the asphalt matrix [13,14]. This incompatibility inevitably leads to severe phase separation during high-temperature storage and excessively high construction viscosity [15,16]. To comprehensively address these issues, desulfurized rubber powder (DRP) has been introduced. Through various thermo-mechanical or chemical activation processes, the S-S and C-S bonds of waste rubber are partially cleaved, restoring the activity of the rubber molecular chains [17,18]. Extensive research has demonstrated that DRP exhibits significantly improved compatibility and interfacial interaction with asphalt, thereby enhancing the storage stability and workability [19]. Nevertheless, while DRP mitigates the compatibility issue, the comprehensive pavement performance of single-DRP-modified asphalt under extreme climatic conditions—particularly its high-temperature modulus and dynamic rutting resistance—often falls short of the rigorous requirements for heavy-traffic highways [20]. Consequently, incorporating secondary polymer modifiers to construct a robust composite modification system has become a widely adopted trend [21].

Waste plastics and elastomers are frequently employed as co-modifiers to enhance the comprehensive performance of CRMA. However, the term “plastics” encompasses a vast variety of polymeric materials with drastically different molecular structures, primarily categorized into plastomers (e.g., Polyethylene, PE) and thermoplastic elastomers (e.g., styrene–isoprene–styrene (SIS), styrene–ethylene–butylene–styrene (SEBS), and styrene–butadiene–styrene (SBS)) [22,23]. For instance, Zhao has reported that the incorporation of semi-crystalline polyolefins like PE can dramatically enhance the high-temperature stiffness and rutting resistance of asphalt [24]. However, this improvement is often achieved at the severe expense of low-temperature cracking resistance and storage stability, as the non-polar PE readily segregates from the polar asphalt matrix [25]. In contrast, block copolymers such as SBS have been heralded as the most successful asphalt modifiers globally. Studies show that SBS can absorb the light aromatic components in asphalt and swell to form a continuous three-dimensional elastic network, providing an excellent balance of high- and low-temperature properties [26,27].

Given the distinct advantages and inherent limitations of different polymers, formulating rubber–plastic composite-modified asphalt has attracted significant academic attention. Studies have attempted to combine rubber with various plastics to achieve synergistic enhancements [28,29]. Peng has blended PE with crumb rubber, noting that rubber can partially alleviate the extreme brittleness caused by PE, though high-temperature storage stability remains a critical concern [30]. Studies also have focused extensively on SBS/rubber composite systems, demonstrating that the physical entanglement between the swollen SBS network and the rubber particles can significantly elevate the overall rheological performance and fatigue life of the asphalt [31,32]. Despite these remarkable advancements, a critical gap remains. Most existing studies tend to evaluate specific, isolated combinations under varying experimental conditions, making direct comparisons highly challenging. A systematic, parallel comparative study on how different molecular structures of plastics—ranging from highly crystalline plastomers (PE) to thermoplastic elastomers with distinct unsaturated (SBS, SIS) or saturated (SEBS) mid-blocks—interact specifically with DRP in the asphalt matrix is still profoundly lacking. Furthermore, the fundamental mechanisms governing the phase compatibility, micro-network evolution, and physical–chemical interactions remain ambiguous.

Meanwhile, the macroscopic pavement performance of composite-modified asphalt mixtures is heavily dependent on the engineering preparation processes, primarily categorized into the “dry method” (adding modifiers directly to aggregates during mixing) and the “wet method” (pre-blending and swelling modifiers within the asphalt) [33,34]. While the dry method offers operational simplicity and lower initial equipment costs, the wet method generally ensures superior polymer swelling and network formation [35]. However, how the distinct molecular architectures of different plastics interact with DRP under these two contrasting preparation methods to ultimately influence the pavement performance is rarely systematically evaluated.

Undeniably, studies have been devoted to the traditional modification of asphalt with conventional polyethylene or crushed rubber [36]. Despite these remarkable advancements, a critical gap remains regarding the fundamental structure–property relationships in multi-polymer composite systems. Therefore, the core novelty of this study lies in three aspects. First, unlike traditional inert crumb rubber, this study utilizes activated DRP to enhance chemical interactions. Second, rather than evaluating an isolated combination, this research provides a systematic, parallel comparison to fundamentally uncover how distinct plastic molecular architectures—ranging from highly crystalline PE to thermoplastic elastomers with distinct unsaturated (SBS, SIS) or saturated (SEBS) mid-blocks—interact specifically with DRP to guide micro-network evolution. Third, it comprehensively bridges the gap between asphalt-level micro-mechanisms and mixture-level macroscopic pavement performance by comparatively evaluating both dry and wet preparation methods.

This study aims to systematically investigate the differential impacts of four representative plastics (PE, SIS, SEBS, and SBS) on the performance and modification mechanism of DRP-modified asphalt. Initially, the basic physical properties and aging resistance of the composite-modified asphalts were evaluated. Subsequently, high- and low-temperature rheological behaviors were profoundly analyzed. Furthermore, the internal phase compatibility, micro-morphological evolution, and chemical interactions were revealed through Fluorescence Microscopy (FM), Scanning Electron Microscopy (SEM), and Fourier Transform Infrared Spectroscopy (FTIR). Finally, the high-temperature rutting resistance, low-temperature flexibility, and moisture susceptibility of the asphalt mixtures, prepared via both dry and wet methods, were comparatively evaluated. The findings of this study are expected to elucidate the structure–property relationships of multi-polymer composite-modified asphalt, providing a crucial theoretical foundation and practical engineering guidance for the targeted selection, process optimization, and sustainable application of diverse waste plastics and tires in high-performance pavement engineering.

## 2. Materials and Methods

### 2.1. Materials

#### 2.1.1. Base Asphalt

90# base asphalt (BA) was selected, and its basic properties are shown in Table 1.

#### 2.1.2. Plastics

**Figure 1:**
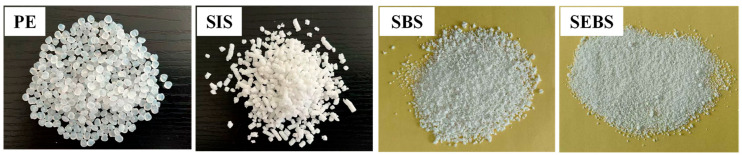
Appearance of different plastics.

PE, SEBS, SIS, and SBS were selected as the raw materials. PE was selected as a representative semi-crystalline polyolefin plastic, while SEBS, SIS, and SBS were chosen as typical styrenic block copolymers with different elastomeric mid-blocks. Their differences in crystallinity, molecular architecture, and elastic characteristics allow the investigation of how plastic type influences the modification mechanism and performance of rubber–plastic composite-modified asphalt [38]. The appearance of different plastics is shown in Figure 1, and the basic properties of different plastics are shown in Table 2.

#### 2.1.3. Rubber Powder

**Figure 2:**
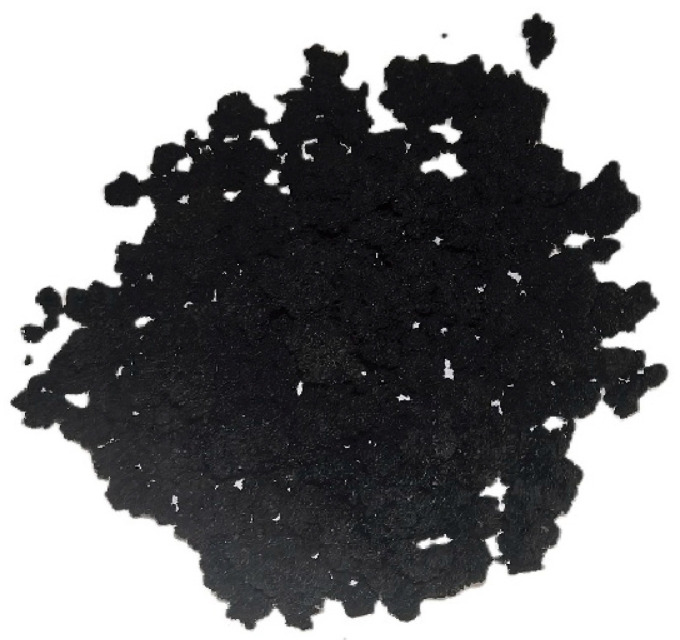
Appearance of DRP.

DRP is prepared from 40-mesh waste tire rubber powder by activation desulfurization through a novel micro-oxygen non-additive desulfurization technology [15]. The appearance of DRP is shown in Figure 2, and the basic properties of different plastics are shown in Table 3.

#### 2.1.4. DRP–Plastic Composite Modifier

To overcome the inherent thermodynamic incompatibility between the diverse plastic phases and the cross-linked rubber network, an intensive thermo-mechanical compatibilization strategy was adopted. Specifically, a screw extruder (HUBEIANJIELUQIAO, Ezhou, China) was used to shear, extrude, and knead the two materials at a high temperature of 200–220 °C. The strong thermo-mechanical shearing forces generated during the twin-screw extrusion process effectively break down the DRP agglomerates, significantly reduce the domain size of the dispersed phase, and promote forced macromolecular chain entanglement and deep physical blending at the phase interfaces [12,39]. This extrusion-induced compatibilization process is essential to preparing a stable DRP–plastic composite modifier. Based on preliminary experimental verification, the composite modifiers determined in this study are shown in Table 4.

It should be noted, however, that the high-temperature extrusion (200–220 °C) poses a risk of thermal–oxidative degradation, particularly for unsaturated block copolymers like SBS and SIS, potentially leading to chain scission and radical generation. In this study, the residence time in the extruder was kept to a strict minimum to mitigate severe degradation without introducing additional chemical variables. Nevertheless, the generated mechanochemical radicals might slightly alter the polymer structure and influence its ultimate interaction with the asphalt.

### 2.2. Test Methods

#### 2.2.1. Preparation of DRP–Plastic Composite-Modified Asphalt

**Figure 3:**
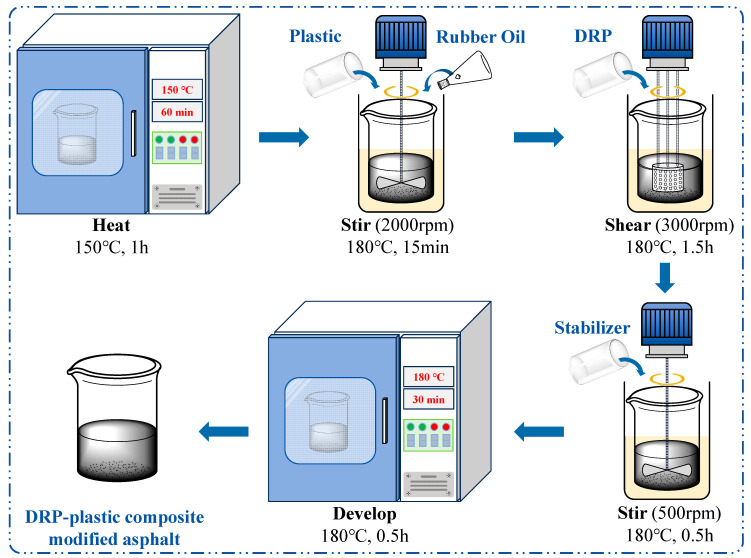
Preparation process of DRP–plastic composite-modified asphalt.

The preparation process of DRP–plastic composite-modified asphalt is shown in Figure 3. The BA is kept at 150 °C for 60 min. The temperature is then raised to 180 °C, and rubber oil and plastic are added and stirred for 15 min at 2000 rpm. Subsequently, the blend was subjected to high-speed shearing. To precisely define the mixing intensity, a laboratory high-shear mixer (JRJ300-YSH, Shanghai Specimen Mold Factory, Shanghai, China), equipped with a standard emulsion stator screen, was utilized. The diameter of the rotor was 70 mm, and the process was conducted in a 1000 mL cylindrical aluminum container with an inner diameter of approximately 120 mm. Subsequently, shearing was performed for 90 min at 180 °C and 3000 rpm. Then, stirring was performed at 500 rpm and 180 °C for 30 min. Finally, developing was performed at 180 °C for 30 min to finish the preparation of DRP–plastic composite-modified asphalt. The asphalts prepared with the modifier in Table 2 were named PE/DRP composite-modified asphalt (PE-DRA), SIS/DRP composite-modified asphalt (SIS-DRA), SEBS/DRP composite-modified asphalt (SEBS-DRA), and SBS/DRP composite-modified asphalt (SBS-DRA), respectively.

#### 2.2.2. Basic Property Tests

To compare the influence of plastic differences on the basic properties of composite-modified asphalt, according to ASTM D5, ASTM D3104, and ASTM D113, the basic properties of the different asphalts, including penetration (Pen), softening point (SP), and ductility (Duct), were measured.

#### 2.2.3. Segregation Test

Storage tests were conducted to determine the asphalt’s segregation and subsequently analyze the storage stability. The samples were placed in an oven at 163 °C for 48 h, followed by freezing in a refrigerator for 4 h. The difference in softening point was then measured.

#### 2.2.4. Rotating Film Oven Test (RTFOT)

To simulate the short-term aging of asphalt during construction and analyze the effects of different plastics on the anti-aging properties of modified asphalt, RTFOT was performed on different modified asphalts. The aging time was 5 h, and the temperature was 163 °C.

#### 2.2.5. Rheological Characterization Tests

(1) Viscosity test

To analyze the fluidity of different modified asphalts, the viscosity of the modified asphalts was tested using a rotational viscometer at temperatures ranging from 130 °C to 180 °C, according to ASTM D4402.

(2) Temperature scanning test

Temperature scanning tests can evaluate the rutting resistance of asphalt at high temperatures. This study conducted temperature scanning tests on different modified asphalts using a Dynamic Shear Rheometer (DSR), with test temperatures ranging from 52 °C to 88 °C and a frequency of 10 rad/s.

(3) Bending beam rheological (BBR) test

The BBR test was employed in this study to evaluate the low-temperature rheological performance of different asphalts. The test was conducted at temperatures of −12 °C to −24 °C.

#### 2.2.6. Scanning Electron Microscopy (SEM)

To analyze the influence of modifier surface morphology on the performance of modified asphalt, SEM was used to characterize the surface features of different composite modifiers and modified asphalts at magnifications of 200×, 500×, and 1000×.

#### 2.2.7. Fluorescence Microscopy (FM)

To observe the dispersion of different modifiers in asphalt and analyze the effect of plastic on the compatibility of modified asphalt, FM (Changheng Rongchuang Technology Co., Ltd., Beijing, China) was used to observe the compatibility of different modified asphalts at magnifications of 10×, 40×, and 100×.

#### 2.2.8. Fourier Transform Infrared Spectroscopy (FTIR)

FTIR analysis can be used to examine the distribution of functional groups of asphalt, thereby further analyzing the changes in the chemical molecular structure of the modified asphalt. A Fourier Transform Infrared Spectrometer was employed to analyze different modified asphalts. The test wavenumber range was set from 4000 to 400 cm^−1^, with 32 scans and a resolution of 4 cm^−1^.

#### 2.2.9. Pavement Performance Tests

To verify the application of DRP–plastic composite asphalts in engineering, the AC-13 asphalt mixture was selected. AC-13 was chosen because it is currently the most commonly used gradation for surface asphalt mixtures. According to the Strategic Highway Research Program, the wheel tracking tests, three-point bending test, immersion Marshall tests, and freeze–thaw splitting tests were conducted. The optimal asphalt–aggregate ratio for AC-13 has been determined through tests, as follows: 4.89 wt% PE-DRP, 5.32 wt% SIS-DRA, 4.91 wt% SEBS-DRP, 5.33 wt% SBS-DRP.

## 3. Results and Discussion

The primary purpose of incorporating the DRP–plastic composite modifiers into the base asphalt is to comprehensively upgrade its service performance to meet the stringent demands of modern heavy-traffic pavements. Specifically, the modification aims to significantly enhance the high-temperature rutting resistance (reflected by an increased softening point and complex modulus), improve the thermal–oxidative aging resistance, and maintain or enhance low-temperature flexibility and stress relaxation capabilities. The following subsections systematically evaluate how the distinct molecular architectures of the four selected plastics interact with DRP to achieve these overarching engineering objectives.

### 3.1. Analysis of Basic Properties

**Figure 4:**
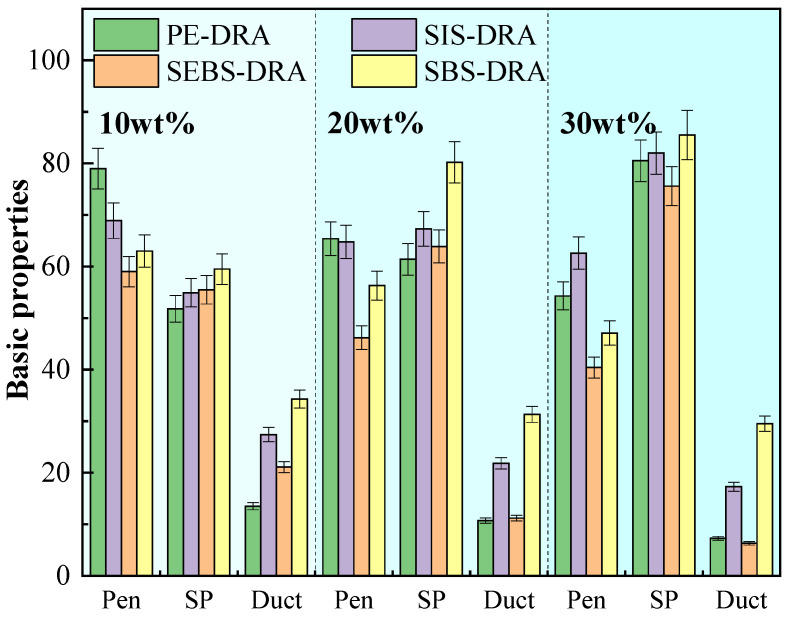
Basic properties of different asphalts.

The basic properties (penetration (Pen), softening point (SP), and ductility (Duct)) evaluate the hardness, high-temperature stability, and low-temperature flexibility of the DRP–plastic composite-modified asphalts, as shown in Figure 4.

As the composite modifier increases from 10 wt% to 30 wt%, the penetration of all asphalts continuously decreases. This indicates that the incorporation of plastics and DRP progressively increases the hardness of the base asphalt. This is primarily due to the swelling effect, wherein the polymers absorb light components in the asphalt, restricting molecular movement. SEBS-DRA exhibits the lowest penetration, suggesting its specific molecular architecture imparts greater rigidity to the matrix. Regarding the softening point, a positive correlation is observed between the modifier and high-temperature performance. SBS-DRA consistently demonstrates the highest softening point, reaching nearly 85 °C at 30 wt%. This exceptional deformation resistance is attributed to the strong synergistic effect between the SBS block copolymer and DRP, which facilitates the formation of a robust three-dimensional spatial cross-linking network [40]. PE-DRA also exhibits a substantial increase, mainly because the crystallization of the semi-crystalline PE acts as physical cross-linking points, enhancing thermal stability.

The most striking differences between the four plastics are reflected in the ductility. While ductility generally decreases with higher dosages, SBS-DRA maintains an excellent ductility of 29 cm even at 30 wt%, indicating superior low-temperature stress relaxation capability. SIS-DRA also maintains acceptable flexibility at 20 wt%. This is because both SBS and SIS possess highly elastic rubbery mid-blocks, imparting excellent toughness to the composite system. Conversely, the ductility of PE-DRA and SEBS-DRA plummets severely at higher dosages. The lack of elastic segments in PE makes the composite asphalt highly brittle at low temperatures. For SEBS-DRA, the saturated nature of its hydrogenated mid-block significantly increases stiffness and restricts polymer chain mobility at 5 °C. Consequently, the SBS-DRP composite modifier achieves the most optimal balance, remarkably enhancing high-temperature stability while preserving excellent low-temperature crack resistance.

Crucially, from an engineering application perspective, the properties of the optimal 20 wt% SBS-DRA formulation strictly comply with the commercial requirements for polymer-modified asphalt specified in the Chinese Technical Specifications for Construction of Highway Asphalt Pavements (JTG F40) [41]. Specifically, its softening point (79.6 °C) and 5 °C ductility (31.4 cm) far exceed the standard commercial thresholds (≥60 °C and ≥20 cm, respectively). This confirms that rather than deteriorating the original BA, the synergistic composite modification successfully upgrades the asphalt to meet heavy-traffic engineering standards.

### 3.2. Analysis of Storage Stability

**Figure 5:**
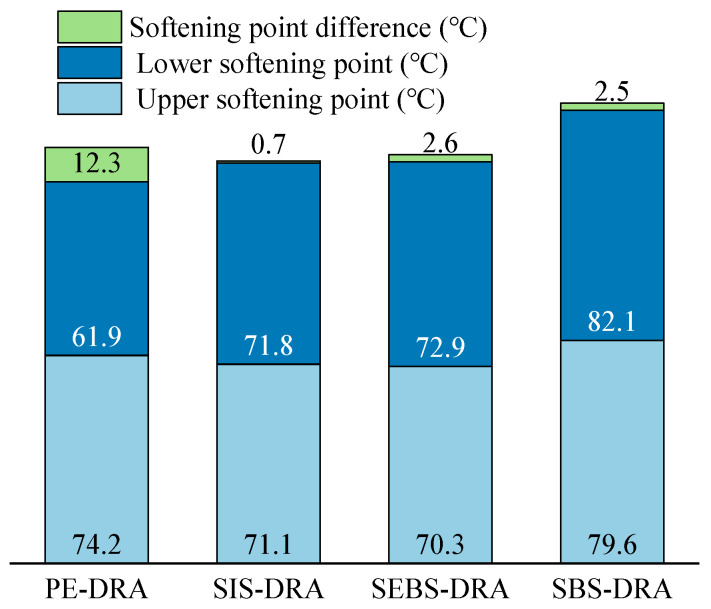
Storage stability of different asphalts.

The high-temperature storage stability of the composite-modified asphalts at a fixed 20 wt% dosage was evaluated using the softening point difference (ΔTR&B) between the top and bottom sections of an aluminum tube, as illustrated in Figure 5. A smaller ΔTR&B indicates better compatibility between the modifiers and the asphalt matrix, with a difference of less than 2.5 °C generally considered acceptable for engineering applications.

The type of plastic significantly affects the storage stability of the composite-modified asphalt. PE-DRA exhibits the most severe phase separation, with a ΔTR&B reaching 12.3 °C, which far exceeds the standard limit. This severe segregation is primarily attributed to the vast difference in polarity and density between the non-polar, semi-crystalline polyethylene and the asphalt matrix. During high-temperature thermal storage, the PE and DRP particles are prone to agglomeration and rapid phase separation from the asphalt phase. Conversely, the composite asphalts modified with block copolymers (SIS, SEBS, and SBS) demonstrate significantly improved storage stability. SIS-DRA shows excellent compatibility, displaying the lowest ΔTR&B of 0.7 °C. The ΔTR&B values for SBS-DRA and SEBS-DRA are 2.5 °C and 2.6 °C, respectively, which are at or marginally above the critical threshold, indicating acceptable stability at the 20 wt% dosage level. This substantial improvement is mainly due to the better thermodynamic compatibility of their rubbery mid-blocks (such as isoprene and butadiene) with the light components in asphalt. Furthermore, these block copolymers can interact synergistically with DRP to form a stable spatial network structure [42]. This network effectively restricts the migration and sedimentation of modifier particles, thereby mitigating macroscopic phase separation.

### 3.3. Analysis of Anti-Aging Performance

**Figure 6:**
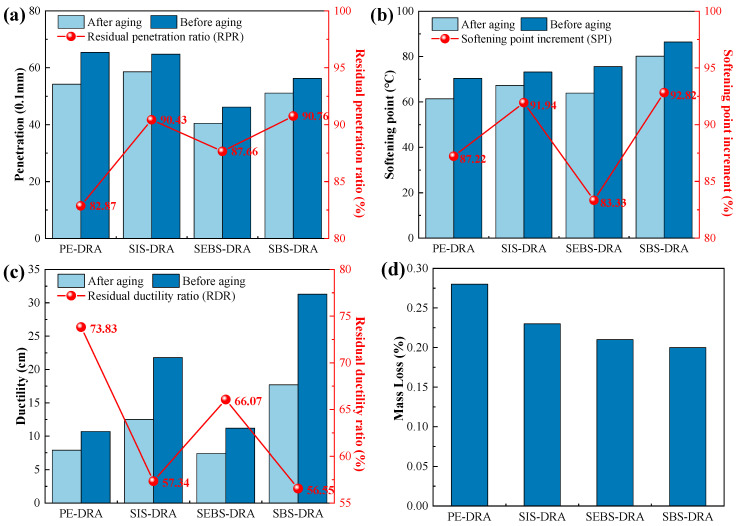
Anti-aging performance of different asphalts: (**a**) Penetration; (**b**) softening point; (**c**) ductility; (**d**) Mass loss.

The short-term thermal–oxidative aging resistance of the composite-modified asphalts was evaluated using RTFOT. The variations in mass loss, residual penetration ratio (RPR), softening point increment (SPI), and residual ductility ratio (RDR) are presented in Figure 6.

As illustrated in Figure 6a, the mass losses of all samples are well below 0.3%, indicating minor volatilization of light components during aging. Notably, SBS-DRA exhibits the lowest mass loss (0.2%). This suggests that the dense spatial network formed by the interaction between SBS and DRP effectively entraps the light oils within the asphalt matrix, preventing their evaporation. Following RTFOT aging, asphalt typically hardens, reflected by a decreased penetration and an increased softening point. SBS-DRA and SIS-DRA demonstrate superior aging resistance, maintaining high RPRs (90.76% and 90.43%, respectively) and displaying high SPI (92.82% and 91.94%). This excellent performance is attributed to the robust polymer–rubber cross-linking network, which acts as a barrier, shielding the asphalt from oxygen intrusion and thermal degradation [43]. Conversely, SEBS-DRA exhibits the lowest SPI (83.33%), and PE-DRA shows the lowest RPR (82.87%), indicating severe aging-induced hardening.

Regarding low-temperature flexibility shown in Figure 6c, the RDRs of SBS-DRA and SIS-DRA (56.55% and 57.34%) are seemingly lower than those of PE-DRA and SEBS-DRA. This phenomenon is primarily due to the unsaturated double bonds present in the polybutadiene and polyisoprene mid-blocks of SBS and SIS, which are susceptible to thermal–oxidative chain scission. However, it is crucial to note that despite the higher percentage loss, the absolute post-aging ductility of SBS-DRA (17.7 cm) still significantly outperforms that of PE-DRA (7.9 cm) and SEBS-DRA (7.4 cm). The higher RDRs of PE and SEBS are largely mathematical artifacts stemming from their extremely poor initial ductility. Overall, SBS-DRA maintains the optimal balance of physical properties after RTFOT, demonstrating outstanding short-term aging resistance.

### 3.4. Analysis of Rheological Performance

#### 3.4.1. Flowability

**Figure 7:**
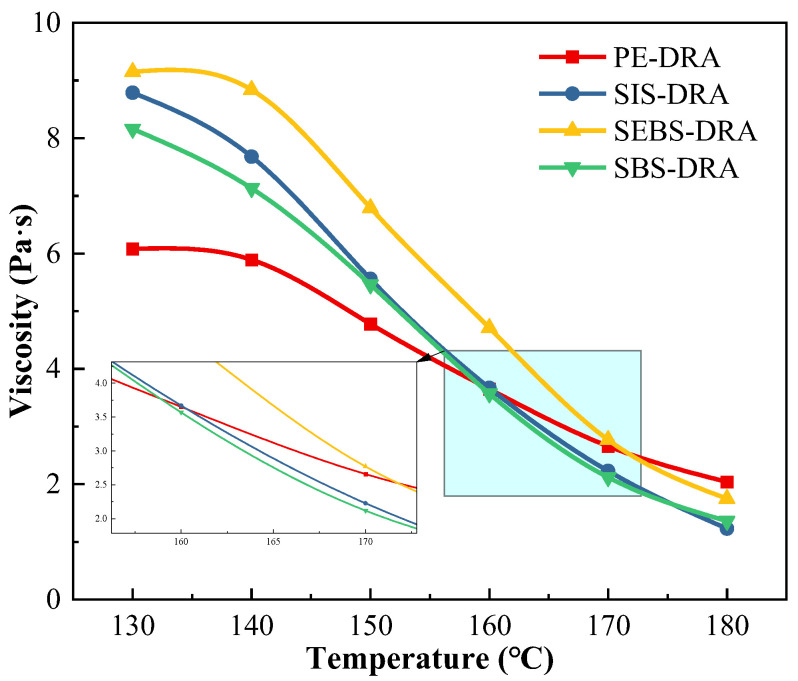
Viscosity–temperature characteristics of different asphalts.

Rotational viscosity is a crucial parameter for evaluating the flowability, pumpability, and construction workability of modified asphalt. The viscosity–temperature curves of the composite-modified asphalts from 130 °C to 180 °C are presented in Figure 7.

The viscosities of all modified asphalts exhibit a continuous downward trend with increasing temperature. This phenomenon is driven by the intensified thermal motion of asphalt molecules and the volume expansion of the matrix at higher temperatures, which reduces internal flow resistance. However, the type of plastic significantly alters the initial viscosity and the temperature susceptibility. Under 140 °C, SEBS-DRA and SIS-DRA display notably high viscosities, with SEBS-DRA reaching 9.15 Pa·s at 130 °C. This high resistance to flow is primarily attributed to the rigidity of the hydrogenated mid-block in SEBS and the intact spatial network formed with DRP. Conversely, PE-DRA exhibits the lowest initial viscosity of 6.08 Pa·s at 130 °C.

A distinct viscosity reversal occurs as the temperature rises to 180 °C. The viscosities of the block copolymer-modified asphalts (SBS, SIS, and SEBS) experience a sharp decline, with SBS-DRA and SIS-DRA dropping significantly to 1.36 Pa·s and 1.23 Pa·s, respectively. This rapid reduction is highly favorable for actual construction processes (pumping, mixing, and compaction). Mechanistically, this occurs because the physical cross-linking nodes formed by the polystyrene end-blocks soften and dissociate at high temperatures, disrupting the polymer network and drastically reducing flow resistance. In sharp contrast, PE-DRA exhibits a much flatter viscosity–temperature curve and maintains the highest viscosity of 2.04 Pa·s at 180 °C. The absence of temperature-sensitive physical cross-linking nodes in the semi-crystalline PE, combined with the severe agglomeration of PE and DRP particles (as confirmed by the storage stability test), persistently increases the internal friction of the fluid even at extreme temperatures. Consequently, SBS-DRA and SIS-DRA demonstrate superior high-temperature workability due to their favorable temperature-responsive network dissociation.

#### 3.4.2. High-Temperature Rheological Performance

**Figure 8:**
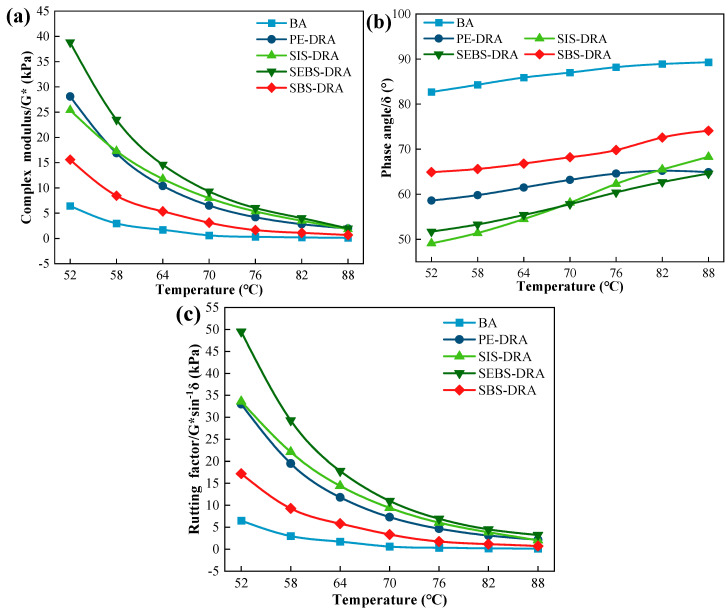
High-temperature rheological performance of different asphalts: (**a**) Complex shear modulus (G*); (**b**) phase angle (δ); (**c**) rutting factor (G*/sinδ).

The high-temperature rheological properties of the base asphalt (BA) and composite-modified asphalts were evaluated. The complex modulus (G*), phase angle (δ), and rutting factor (G*/sinδ) from 52 °C to 88 °C are presented in Figure 8. The higher G*, higher rutting factor, and lower δ show better high-temperature performance in anti-rutting.

As temperature increases, the thermal motion of asphalt molecules intensifies, resulting in a continuous decrease in G* and G*/sinδ, and an increase in δ for all samples. Compared to BA, all composite-modified asphalts exhibit significantly higher G* and lower δ. This demonstrates that the incorporation of DRP and plastics effectively transforms the asphalt from a predominantly viscous fluid into a highly elastic viscoelastic material, which is attributed to the formation of a spatial polymer–rubber cross-linking network that resists shear deformation. Significant differences are observed among the four plastic modifiers. SEBS-DRA exhibits the highest G* and G*/sinδ across the entire temperature range, with its rutting factor reaching 49.5 kPa at 52 °C. This exceptional dynamic rutting resistance is primarily driven by the saturated, hydrogenated mid-blocks of SEBS, which impart substantial rigidity and thermal stability to the asphalt matrix under dynamic loading. SIS-DRA and PE-DRA also demonstrate robust high-temperature deformation resistance, outperforming SBS-DRA in terms of dynamic shear stiffness. While SBS-DRA displays the lowest G*/sinδ among the modified asphalts (17.2 kPa at 52 °C), it remains vastly superior to BA (6.5 kPa). This relatively lower stiffness under intermediate-to-high temperatures is actually consistent with its exceptional low-temperature ductility observed earlier. The unsaturated polybutadiene segments in SBS provide a softer, more flexible network than the saturated SEBS, preventing the asphalt from becoming excessively brittle. Therefore, while SEBS-DRA provides the maximum rigidity against high-temperature rutting, SBS-DRA maintains a more balanced viscoelastic profile, preventing over-stiffening while still satisfying high-temperature performance requirements.

#### 3.4.3. Low-Temperature Rheological Performance

**Figure 9:**
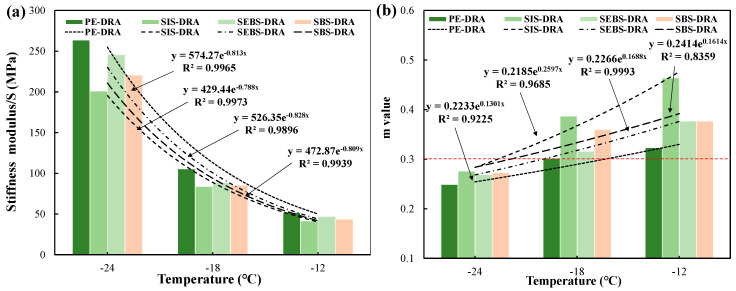
Low-temperature rheological performance of different asphalts: (**a**) Stiffness modulus; (**b**) m value.

The low-temperature cracking resistance of the composite-modified asphalts was evaluated. The creep stiffness (S) and creep rate (m-value) at −12 °C, −18 °C, and −24 °C are presented in Figure 9. Generally, a lower S value indicates better flexibility, while a higher m-value suggests superior stress relaxation capability. The Strategic Highway Research Program (SHRP) specifies S ≤ 300 MPa and m ≥ 0.3 for satisfactory low-temperature performance.

As the temperature drops from −12 °C to −24 °C, the S values of all asphalts increase significantly while their m-values decrease, reflecting the transition of the asphalt from a viscoelastic state to a brittle glassy state. However, the varying molecular structures of the incorporated plastics result in distinct low-temperature rheological behaviors. Among the composite-modified asphalts, SBS-DRA and SIS-DRA exhibit noticeably superior low-temperature performance, characterized by lower S values and higher m-values. This excellent low-temperature flexibility is fundamentally derived from the highly elastic rubbery mid-blocks (polybutadiene in SBS and polyisoprene in SIS). These flexible polymeric chains maintain high mobility even at sub-zero temperatures, effectively dissipating thermal shrinkage stresses and resisting crack propagation. In sharp contrast, SEBS-DRA and PE-DRA display significantly higher stiffness and poorer relaxation capacities. SEBS-DRA shows the highest S value and lowest m-value at lower temperatures. While the hydrogenation of the mid-block in SEBS substantially enhances its high-temperature rutting resistance (as confirmed in the DSR analysis), it simultaneously increases the rigidity of the polymer chains, severely restricting their conformational movement at low temperatures. Similarly, the lack of elastic segments and the semi-crystalline nature of PE make PE-DRA highly brittle, leading to deficient stress relaxation. These BBR findings are highly consistent with the macroscopic ductility results, further confirming that the SBS-DRP composite modifier provides the optimal low-temperature crack resistance.

### 3.5. Analysis of Microscopic Morphology

#### 3.5.1. Analysis of Surface Morphology

(1) Composite modifier

**Figure 10:**
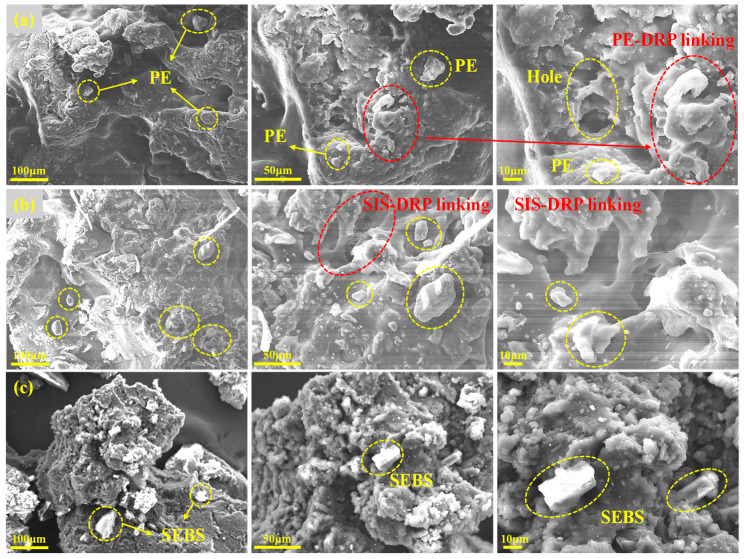

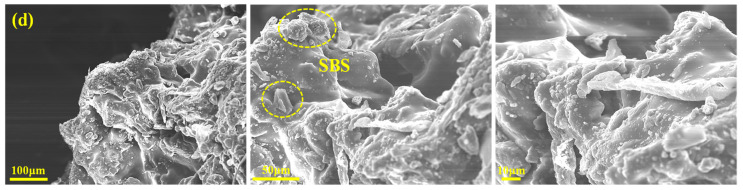
Surface morphology of different composite modifiers: (**a**) PE-DRP; (**b**) SIS-DRP; (**c**) SEBS-DRP; (**d**) SBS-DRP.

SEM was utilized to observe the surface micro-morphology of the four composite modifiers, as shown in Figure 10. These structural differences provide fundamental insights into their macroscopic performance variations in the asphalt matrix.

As shown in Figure 10a, the PE-DRP composite exhibits a distinct granular morphology with sharp phase boundaries and discrete particles. This indicates a purely physical blending state with poor interfacial adhesion, stemming from the significant polarity difference between the semi-crystalline PE and DRP. This fragmented microstructure fundamentally explains the severe storage segregation and poor low-temperature ductility of PE-DRA.

For the block copolymer-based modifiers, interfacial compatibility is notably improved. In Figure 10c, SEBS-DRP displays a relatively integrated but somewhat rigid and textured surface, reflecting the stiffening effect of its saturated hydrogenated mid-blocks. This structural rigidity contributes to its superior high-temperature rutting resistance but compromises low-temperature flexibility. Figure 10b demonstrates that SIS-DRP forms a more cohesive structure with slight surface wrinkles, indicating enhanced mutual interaction. Remarkably, as depicted in Figure 10d, the SBS-DRP composite presents a highly continuous, homogeneous, and dense three-dimensional spatial network. The boundaries between SBS and DRP are almost completely blurred, suggesting deep swelling, excellent compatibility, and strong physical entanglement of the polymer chains. This robust, interwoven microstructure is the primary reason the SBS-DRP modifier imparts optimal storage stability, exceptional ductility, and balanced viscoelastic properties to asphalt [44].

(2) Composite asphalt

**Figure 11:**
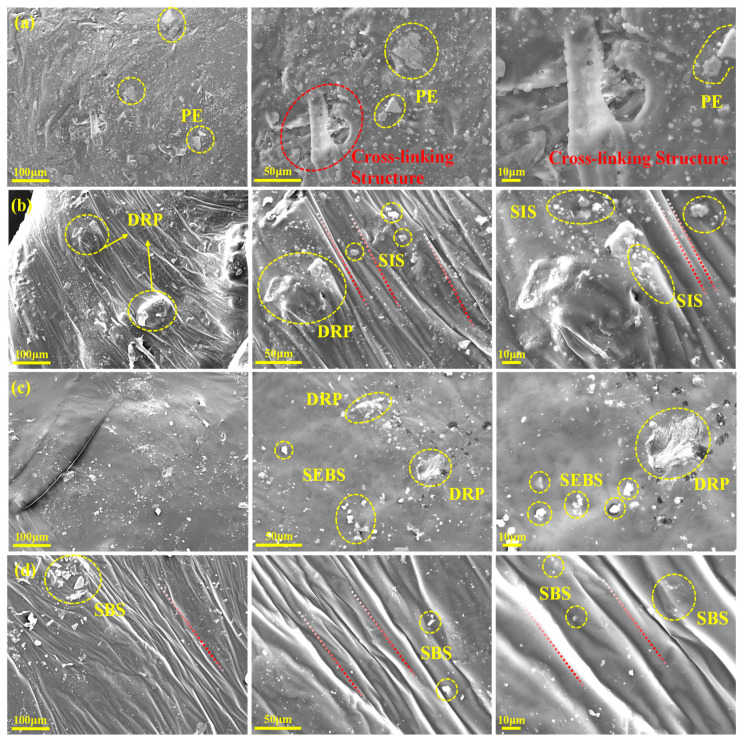
Surface morphology of different asphalts: (**a**) PE-DRA; (**b**) SIS-DRA; (**c**) SEBS-DRA; (**d**) SBS-DRA.

To further investigate the dispersion state and compatibility of the composite modifiers within the asphalt matrix, the micro-morphologies of the four composite-modified asphalts were observed using SEM, as presented in Figure 11. The phase structure and interfacial interaction directly determine the macroscopic properties of the asphalt.

As illustrated in Figure 11a, PE-DRA exhibits a highly heterogeneous two-phase structure. The modifiers are severely agglomerated, displaying distinct, sharp interfacial boundaries with the surrounding asphalt matrix. This pronounced phase separation confirms the poor thermodynamic compatibility between the non-polar, semi-crystalline polyethylene and the asphalt. Because the modifier particles simply act as isolated inclusions rather than forming a continuous network, this fundamentally explains the severe macroscopic segregation observed in the storage stability test and its high brittleness at low temperatures.

For the asphalts modified with block copolymers, the interfacial compatibility is significantly improved, yet structural differences remain. In Figure 11c, SEBS-DRA shows a relatively continuous but noticeably rough and textured surface with some localized rigid domains. This indicates that while SEBS interacts with the asphalt, its saturated, hydrogenated mid-blocks restrict complete homogeneous swelling. This microscopic rigidity contributes to its exceptionally high complex modulus at high temperatures but limits its stress relaxation capability at low temperatures [45]. Figure 11b demonstrates that SIS-DRA presents a more uniform and cohesive morphology than PE-DRA and SEBS-DRA, owing to the better compatibility of the isoprene segments with the light components of asphalt.

Remarkably, as shown in Figure 11d, SBS-DRA presents an exceptionally smooth, highly continuous, and homogeneous microscopic morphology. The boundaries between the SBS-DRP composite modifier and the asphalt matrix are almost entirely blurred. This indicates that the modifier has been deeply swollen, highly dispersed, and perfectly integrated into the matrix. This microstructural evolution provides direct visual evidence for the formation of a dense, stable, and interwoven three-dimensional polymer–rubber spatial network. It is this perfectly integrated microstructure that endows SBS-DRA with outstanding storage stability, superior low-temperature ductility, and well-balanced rheological properties.

#### 3.5.2. Analysis of Compatibility

**Figure 12:**
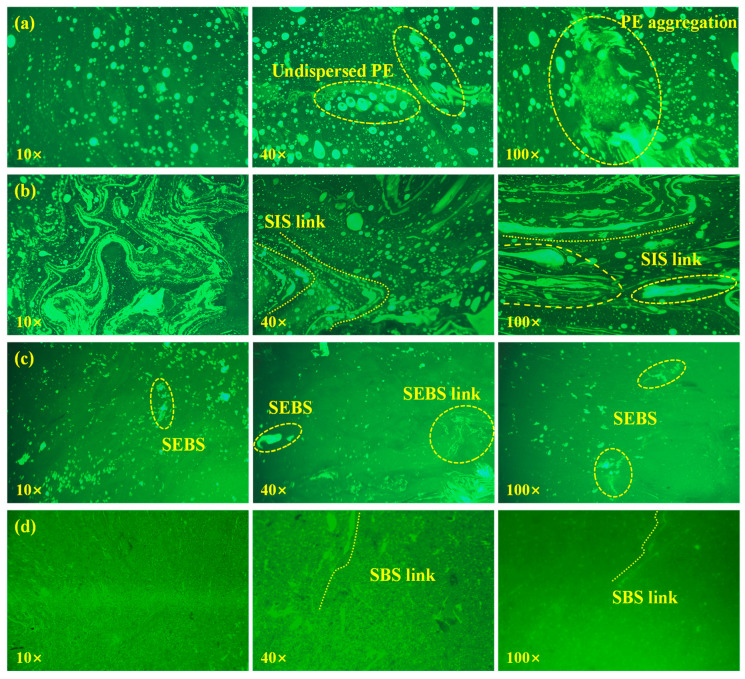
Interface compatibility of different asphalts: (**a**) PE-DRA; (**b**) SIS-DRA; (**c**) SEBS-DRA; (**d**) SBS-DRA.

FM was employed to visually investigate the phase distribution, polymer network evolution, and internal compatibility of the composite modifiers within the asphalt matrix, as shown in Figure 12. In FM images, the polymer phase absorbs light and emits fluorescence (appearing bright), while the base asphalt phase does not fluoresce (appearing dark).

As shown in Figure 12a, PE-DRA exhibits the poorest compatibility. Across all magnifications (10× to 100×), massive, isolated fluorescent aggregates are clearly visible. The presence of “Undispersed PE” and severe “PE aggregation” indicates that the semi-crystalline polyethylene fails to absorb the light oils in the asphalt for effective swelling. This massive, discontinuous phase separation acts as visual proof of the severe macroscopic segregation and poor low-temperature ductility discussed previously.

In contrast, the block copolymers demonstrate significantly improved dispersion. For SIS-DRA in Figure 12b, the fluorescent phase begins to extend and form discernible “SIS links”, indicating a certain degree of polymer network formation facilitated by the flexible isoprene segments. SEBS-DRA in Figure 12c also exhibits dispersed polymer domains and localized networking (“SEBS links”). However, due to the rigidity of its saturated hydrogenated segments, the fluorescent phase in SEBS-DRA appears somewhat granular and less continuous than that of unsaturated block copolymers, which perfectly explains its high shear stiffness but lower ductility.

Remarkably, SBS-DRA in Figure 12d exhibits the most optimal colloidal structure. At higher magnifications (40× and 100×), the bright fluorescent polymer phase forms a highly fine, continuous, and densely interwoven three-dimensional network (“SBS links”). The boundaries between the polymer phase and the dark asphalt phase are highly blurred and intimately mixed. This indicates that SBS and DRP fully swell and perfectly co-blend with the asphalt matrix. This robust and continuous structural network effectively locks the light components of the asphalt, thoroughly explaining the exceptional storage stability, outstanding low-temperature flexibility, and excellently balanced rheological performance of SBS-DRA [46].

### 3.6. Analysis of Chemical Functional Groups

**Figure 13:**
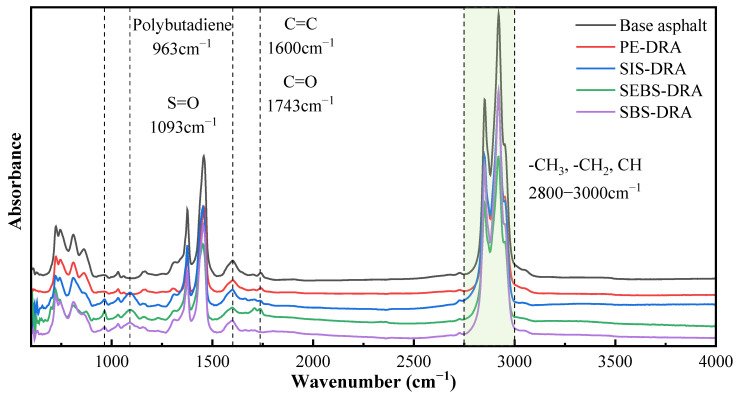
Functional group characterization results of different asphalts.

FTIR was conducted to investigate the functional groups and chemical interactions between the BA and the composite modifiers. The FTIR spectra of BA and the four composite-modified asphalts in the wavenumber range of 4000–500 cm^−1^ are presented in Figure 13.

As depicted in Figure 13, the base asphalt exhibits typical characteristic absorption peaks: the strong peaks at 2920 cm^−1^ and 2850 cm^−1^ correspond to the stretching vibrations of aliphatic C-H bonds, while the peaks around 1460 cm^−1^ and 1375 cm^−1^ are attributed to the bending vibrations of -CH_2_ and -CH_3_ groups. The minor peak near 1600 cm^−1^ is associated with the C=C stretching vibration of aromatic rings. Following the incorporation of the composite modifiers, the overall shapes of the infrared spectra for the modified asphalts remain highly similar to that of BA, with the primary absorption peaks essentially unchanged. However, specific superimposed peaks from the modifiers can be observed. For instance, in the spectra of SBS-DRA, SIS-DRA, and SEBS-DRA, the characteristic absorption peaks near 699 cm^−1^ and 760 cm^−1^ become more pronounced due to the C-H out-of-plane bending vibrations in the polystyrene blocks. Additionally, a specific peak around 966 cm^−1^ appears in SBS-DRA, which corresponds to the trans-CH=CH- bending vibrations from the polybutadiene segments. Crucially, no distinct new absorption peaks emerge in any of the composite-modified asphalts. This infrared spectroscopic evidence demonstrates that the interactions among the plastics, DRP, and the asphalt matrix are primarily characterized by physical blending, swelling, and physical entanglement, without any significant or complex chemical reactions occurring during the preparation process.

### 3.7. Analysis of Pavement Performance

**Figure 14:**
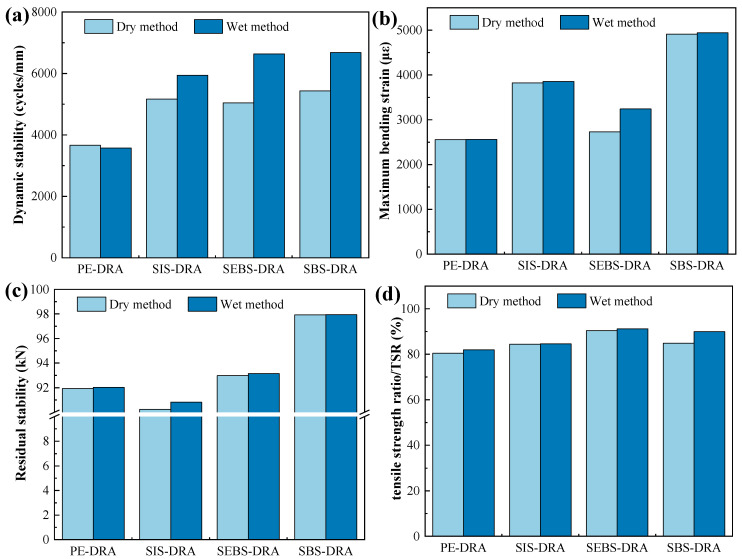
Pavement performance of different asphalt mixtures: (**a**) Wheel tracking test results; (**b**) three-point bending test results; (**c**) immersion Marshall test results; (**d**) freeze–thaw splitting test results.

To verify the practical application potential of the composite modifiers, the high-temperature rutting resistance, low-temperature cracking resistance, and moisture susceptibility of the asphalt mixtures were evaluated. Furthermore, two different preparation processes—the dry method and the wet method—were compared, with the results presented in Figure 14.

Regarding high-temperature performance in Figure 14a, the dynamic stability (DS) of the mixtures modified with block copolymers (SBS, SIS, SEBS) is significantly higher than that of PE-DRA. According to standard specifications (JTG F40), the DS for modified asphalt mixtures under heavy traffic should be no less than 2800 cycles/mm. Notably, the preparation method exerts a profound impact. For SBS-DRA, SEBS-DRA, and SIS-DRA, the wet method yields substantially higher DS values compared to the dry method. Specifically, the DS of SBS-DRA and SEBS-DRA under the wet method reaches 6682 and 6634 cycles/mm, respectively, which is more than double the regulatory requirement. This significant enhancement is attributed to the fact that the wet method allows the polymer and DRP to fully swell, interact, and establish a robust three-dimensional cross-linking network within the asphalt matrix before mixing with aggregates, thereby strongly binding the aggregate skeleton. Conversely, PE-DRA shows negligible improvement with the wet method due to its inherent lack of compatibility and inability to form a continuous network.

The low-temperature cracking resistance, evaluated by the maximum bending strain in Figure 14b, aligns perfectly with BBR and ductility test results. SBS-DRA demonstrates the most outstanding low-temperature flexibility, with its maximum bending strain approaching 5000 under both methods. In sharp contrast, PE-DRA and SEBS-DRA exhibit much lower bending strains, indicating elevated brittleness. The rigid hydrogenated mid-blocks in SEBS and the semi-crystalline nature of PE fail to provide adequate stress relaxation within the mixture at low temperatures. The moisture susceptibility was evaluated through residual stability in Figure 14c and tensile strength ratio (TSR) in Figure 14d. The regulatory standards mandate a residual stability of ≥85% and a TSR of ≥80%. All composite-modified asphalt mixtures meet the general standard requirements. However, SBS-DRA and SEBS-DRA display superior moisture resistance, with the TSR of the wet-method SBS-PMB mixture reaching 90%, comfortably exceeding the specification limit. Furthermore, the wet method consistently enhances moisture resistance across most groups. The pre-formed dense polymer network in the wet method improves the interfacial adhesion between the asphalt and the aggregates, effectively mitigating moisture intrusion and asphalt stripping. In conclusion, the wet preparation method is highly recommended for block copolymer/DRP composite modifiers. Among all evaluated mixtures, SBS-DRA prepared via the wet method exhibits the most exceptional and balanced comprehensive pavement performance.

## 4. Conclusions

Based on the comprehensive evaluation of the physical, rheological, microscopic, and mixture pavement properties of composite asphalts modified with DRP and four different plastics (PE, SIS, SEBS, and SBS), the following main conclusions can be drawn:

(1) The molecular architecture of the plastics fundamentally dictates their compatibility with the asphalt matrix. The non-polar, semi-crystalline PE results in severe phase separation and massive agglomeration. Conversely, SBS interacts synergistically with DRP to form a stable, densely interwoven three-dimensional network, ensuring excellent high-temperature storage stability even at an ultra-high dosage of 30 wt%.
(2) SEBS-DRA demonstrates the highest high-temperature rutting resistance due to the high rigidity of its hydrogenated mid-blocks, but this significantly compromises its low-temperature flexibility. SBS-DRA and SIS-DRA maintain a superior viscoelastic balance, offering excellent low-temperature stress relaxation and ductility while satisfying high-temperature structural requirements.
(3) Microstructural observations (SEM and FM) and FTIR analyses reveal that the modification is primarily a physical blending and swelling process without complex chemical reactions. SBS achieves the deepest swelling and most homogeneous dispersion within the asphalt, effectively locking in the light components and providing the structural basis for its outstanding macroscopic performance.
(4) The pavement performance of block copolymer-modified asphalt mixtures is highly dependent on the preparation process. The wet method significantly outperforms the dry method by allowing sufficient pre-swelling and polymer network development prior to aggregate mixing, which substantially enhances both dynamic stability and moisture resistance.
(5) Among the evaluated materials, the SBS-DRP composite modifier is proven to be the most optimal choice. Particularly when prepared via the wet method, SBS-DRA provides the best comprehensive performance, remarkably improving the high-temperature stability, low-temperature cracking resistance, and overall durability of the asphalt pavements.

Novelty and Limitations: Compared to the extensively published research focusing on conventional single-polymer modifications or simple inert crumb rubber, the core novelty of this study lies in its systematic, parallel elucidation of how distinct plastic molecular architectures (ranging from semi-crystalline plastomers to block copolymers with saturated/unsaturated mid-blocks) specifically interact with activated DRP. This provides a fundamental material-selection framework for multi-source solid waste recycling. However, there are objective limitations to the current application of this study. The performance evaluations are strictly constrained to laboratory-scale conditions, without the incorporation of extrusion-stage antioxidants, and are limited to short-term aging simulations. The long-term durability under complex actual field environments, the atomic-level thermodynamic interaction mechanisms, and the actual construction emission profiles remain uncharacterized. Therefore, before widespread commercial application, future research must focus on constructing full-scale field test sections, employing molecular dynamics (MD) simulations, and conducting rigorous Life Cycle Assessments (LCAs) and VOC emission evaluations.

## Figures and Tables

**Table 1:** Basic properties of BA.

Items		Requirements	Tested Value	Test Method
Penetration (25 °C, 100 g)/0.1 mm		80–100	85	T 0604 [37]
Ductility (10 °C, 5 cm/min)/cm		≥45	>100	T 0605 [37]
Softening point/°C		≥45	45.6	T 0606 [37]
Viscosity (135 °C) (Pa·s)		0.432	≥0.16	T 0625 [37]
Dynamic viscosity (60 °C) (Pa·s)		238.7	≥160	T 0620 [37]
After RTFOT	Mass loss/%	≤±0.8	−0.428	T 0610 [37]
	Penetration/%	≥57	60.4	T 0604 [37]
	Ductility (10 °C)/cm	≥8	9	T 0605 [37]

**Table 2:** Basic properties of plastic.

Materials	Density/g·cm^−3^	Pull-Up Strength/MPa	Elongation at Break/%	Hardness
PE	0.92	18	600	45
SIS	0.93	26.0	1130	40
SBS	0.94	18	650	70
SEBS	0.91	3	400	70

**Table 3:** Basic properties of DRP.

Items	Relative Density	Metal Content/%	Fiber Content/%	Rubber Hydrocarbon/%	Ash Content/%	Acetone Extract/%	Carbon Black/%
Test value	1.21	0.018	0.12	62	5.2	9	34

**Table 4:** DRP–plastic composite modifier and the material proportion.

Composite Modifier	Proportion
PE-DRP	DRP:PE = 1:6
SIS-DRP	DRP:SIS = 3:7
SEBS-DRP	DRP:SEBS = 3:7
SBS-DRP	DRP:SBS = 3:7

## Data Availability

The original contributions presented in this study are included in the article. Further inquiries can be directed to the corresponding authors.
